# SARS-CoV-2 and West Nile Virus Prevalence Studies in Raccoons and Raccoon Dogs from Germany

**DOI:** 10.3390/v14112559

**Published:** 2022-11-19

**Authors:** Markus Keller, Norbert Peter, Cora M. Holicki, Anna V. Schantz, Ute Ziegler, Martin Eiden, Dorian D. Dörge, Andreas Vilcinskas, Martin H. Groschup, Sven Klimpel

**Affiliations:** 1 Friedrich-Loeffler-Institut, Federal Research Institute for Animal Health, Institute of Novel and Emerging Infectious Diseases, Suedufer 10, 17493 Greifswald–Insel Riems, Germany; 2 Institute for Ecology, Evolution and Diversity, Integrative Parasitology and Zoophysiology (IPZ), Goethe-University, Max-von-Laue-Str. 13, 60439 Frankfurt/Main, Germany; 3 Institute of Insect Biotechnology, Justus-Liebig-University of Giessen, Heinrich-Buff-Ring 26-32, 35392 Giessen, Germany; 4 Branch Bioresources, Fraunhofer Institute for Molecular Biology and Applied Ecology, Ohlebergsweg 12, 35392 Giessen, Germany; 5 Senckenberg Biodiversity and Climate Research Centre (SBiK-F) and LOEWE Centre for Translational Biodiversity Genomics (LOEWE TBG), Senckenberganlage 25, 60325 Frankfurt/Main, Germany

**Keywords:** SARS-CoV-2, West Nile virus, wild life reservoir, raccoon, raccoon dog, RT-qPCR, neutralization assay

## Abstract

Unlike farm animals, wild animals are not subject to continuous health surveillance. Individual projects designed to screen wildlife populations for specific pathogens are, therefore, also of great importance for human health. In this context, the possible formation of a reservoir for highly pathogenic zoonotic pathogens is a focus of research. Two of these pathogens that have received particular attention during the last years are the novel severe acute respiratory syndrome coronavirus type 2 (SARS-CoV-2), due to its fast global spread and high impact to the human health, and, since its introduction into Germany, the flavivirus West Nile virus (WNV). Especially in combination with invasive vertebrate species (e.g., raccoons (*Procyon lotor*) and raccoon dogs (*Nyctereutes procyonoides*) in Germany), risk analysis must be done to enable health authorities to assess the potential for the establishment of new wild life reservoirs for pathogens. Therefore, samples were collected from raccoons and raccoon dogs and analyzed for the presence of SARS-CoV-2 and WNV infections in these populations. Molecular biological and serological data obtained imply that no SARS-CoV-2 nor WNV reservoir has been established in these two wild life species yet. Future investigations need to keep an eye on these invasive carnivore populations, especially since the close contact of these animals to humans, mainly in urban areas, would make animal–human transmission a challenge for human health.

## 1. Introduction

Unlike farm animals, wild animals can carry bacteria, parasites and viruses without being quickly noticed. Whereas farm animals are subject to regular monitoring, the monitoring of the health status of wild animals requires special projects that need intensive planning and the use of often considerable financial resources [1,2,3]. For this reason, diseases are often only detected by chance. Exceptions to this are rapidly spreading diseases with conspicuous disease symptoms such as avian influenza [4,5,6] or African swine fever [7,8,9], both of which cause considerable numbers of cases and have severe disease courses. In addition to viral diseases, bacteria and parasites also play a role in the wildlife population that should not be underestimated, as they often also go undetected and untreated [10].

Due to changes in the use of rural areas and the spread of urban structures, as well as the globalization of trade [11,12] and changes in climate [13], there has been a shift in the occurrence of wildlife toward “urbanization” in recent years [14,15]. Wildlife is increasingly in contact with humans and often occurs in urban areas [16]. This not only increases the likelihood that infections—for example, those already mentioned—are transmitted from wild animals to farm animals, but also that pathogens can be passed from humans to wild animals and vice versa [17]. The focus here is on pathogens that find a reservoir in wild animal populations and can remain undetected there for a long time and in the course acquire changes in their genomic nucleic acids. If such a pathogen is then transmitted to humans, it can also spread in the human population and, as a zooanthroponotic pathogen [18,19,20], can lead to severe pandemic outbreaks. On the other hand, human diseases may also pose a major threat to the overall population if transmitted to the wild animal population (anthropozoonoses) [21,22].

Another important aspect of these considerations are new invasive species. These species are characterized by a high degree of adaptability and a rapid geographical spread. They may bring certain pathogens from their areas of origin to the newly colonized areas or may be immune to pathogens present in the area. In addition to the occupation of ecological niches by the newcomers and a pushing back of endemic species, possibly brought-along pathogens [23,24] represent a not insignificant danger for the existing populations [25,26]. Especially if the introduced species prove resistant to these pathogens, the displacement process is accelerated. Conversely, immunity to native pathogens can lead to the emergence of a new reservoir in wildlife that goes unnoticed due to a lack of clinical signs. If animal–human contact then results in transmission of the pathogen, as is considered likely for severe acute respiratory syndrome coronavirus type 2 (SARS-CoV-2 [27,28]), for example, chains of infection can emerge that affect humans as well as wildlife and livestock.

A study conducted during the SARS-CoV-2 pandemic on the possible formation of a wildlife reservoir naturally focuses on the novel coronavirus that first appeared in China and then spread across the globe [21,28,29,30,31,32]. This virus is a single-stranded RNA virus whose origin is most likely in the wildlife population of China. It came into contact with humans through a market where wild animals were also sold and spread very quickly to become a global pandemic [33]. Recent studies have shown that raccoon dogs (*Nyctereutes procyonoides*), which are native to China and invasive to Germany, are highly susceptible to the virus [34,35], and raccoons (*Procyon lotor*), which are native to North America, can also be infected experimentally [36]. The present study includes studies on the presence of SARS-CoV-2 in the wild raccoon population using RT-qPCR [37] assays for SARS-CoV-2-specific nucleic acids and serological assays to detect antibodies against this virus.

In addition to studies on whether raccoons are a wildlife reservoir for SARS-CoV-2, the animal were also tested for antibodies against flaviviruses, a group of viruses that have spread in Germany in recent years with pathogens that are highly pathogenic for humans [38,39,40,41]. While the flavivirus tick-borne encephalitis virus (TBEV) [42,43] has been detected in Germany for almost a hundred years [44], the West Nile virus (WNV) has only been present in Germany since 2018 [45,46]. Both flaviviruses have in common that they can cause severe diseases in humans. In addition to humans, animal populations (including, e.g., horses and birds, depending on the virus) are also affected, in which severe clinical manifestations (neurological signs) and even death can occur. For immunologically healthy humans, the flavivirus Usutu virus (USUV), present in Germany since 2010 [47,48], poses no threat to the majority, while it can cause epidemic collapse of entire bird populations [49,50,51]. All three viruses mentioned are serologically related and belong to the Japanese encephalitis serogroup [52]. Due to the close relationship within a serogroup, cross-reactivity of antibodies against these viruses often occurs, so that serological assays must always test against antigens of the other viruses as well. Among the antibody detection systems used, the serum neutralization test (SNT) has the highest specificity, so that a reliable differentiation between antibodies is possible here. Other assays, such as ELISA, often cannot distinguish between the different viruses due to flavivirus-dependent cross-reactivities [53].

In order to close the research gap regarding the possible formation of new virus reservoirs in the aforementioned invasive species, the studies described here were conducted. For this purpose, blood samples from raccoons and from raccoon dogs from different capture sites in Germany were analyzed by RT-qPCR for SARS-CoV-2 and WNV- specific nucleic acids and for antibodies against SARS-CoV-2 and WNV. The aim of the present study was to clarify via positive findings whether raccoons and raccoon dogs came into contact with the viruses and may have developed virus-specific antibodies.

## 2. Materials and Methods

### 2.1. Sample Collection

Sample design, funding and sampling was conducted within the framework of the ZOWIAC project (Zoonotic and Wildlife Ecological Impacts of Invasive Carnivores) funded by the German Federal Environmental Foundation (DBU) and the Uniscientia Foundation. Between January 2021 and June 2022, blood, tissue and swab samples were collected from a total of 229 raccoons and 11 raccoon dogs and tested for WNV and SARS-CoV-2. The raccoons and raccoon dogs were hunted or trapped. Sampling was carried out in accordance with the applicable legal regulations (§28a Bundesjagdgesetz (Federal Hunting Act, BJagdG) in connection with §40e Bundesnaturschutzgesetz (Federal Nature Conservation Act, BNatSchG)). Throughout the study area, no special permits (other than a general hunting license) were required to legally hunt raccoons and raccoon dogs.

Sampling kits were sent to the participating hunters and consisted of: instruction sheet for sampling, accompanying sheet for data collection, Sigma Virocult^®^ swabs (mwe, Corsham, Wiltshire, UK), Forensic Swab L (Sarstedt, Nümbrecht, Germany), disposable syringe and cannula, three collection vessels (2 × 15 mL with yellow lid, 1 × BD Vacutainer^®^ blood collection tubes Pediatrics Li-Heparin (17 IU/mL) (turquoise) (Becton Dickinson, Plymouth, UK), disposable scalpel, disposable forceps, leak-proof protective bag, nitrile gloves and a face mask.

Immediately after killing the animals, two oropharyngeal swabs were taken using Virocult^®^ swabs and Forensic Swab L. From each animal a blood sample (2 mL) was collected from the opened head or neck vein using a disposable syringe and transferred to the heparinized blood collection tube (BD Vacutainer^®^ Blood Collection Tube Pediatric Li-Heparin). Additionally, 1 cm^3^ tissue sample from the lung and foreleg muscle were collected and each transferred to a 15 mL collection tube containing 7 mL of preservation medium (90% Hanks’ Balanced Salt Solution (VWR, Darmstadt, Germany) + 10% fetal bovine serum, certified, heat inactivated (Gibco™, ThermoFisher, Schwerte, Germany)).

The samples were returned by the hunters to the Institute for Integrative Parasitology and Animal Physiology, Goethe University, in the provided and stamped transport bag. Here, the recording of the origin data and labeling of the samples took place. Muscle samples were stored at −20 °C. The swab samples, blood samples and lung samples were forwarded to the Institute of Novel and Emerging Infectious Diseases, Friedrich Loeffler Institut (FLI), Island of Riems for virus diagnostics. Samples of 205 individuals were tested in all of the assays and are presented in this article, while remaining samples were not tested in all assays due to lack of material.

### 2.2. Molecular Detection of Viral RNA

#### 2.2.1. SARS-CoV-2

For RT-qPCR, dry swabs were incubated in 1 mL of AVL for 30 min to elute the sample material bound and then inactivated at 56 °C for 10 min. From the virus transport medium (VTM) of the ViroCult swabs, 140 µL was transferred to 560 µL of AVL and then also heat inactivated for 10 min as recommended. An approximately 1 mm^3^ piece was taken from the tissue samples and transferred to 600 µL of RLT buffer (RNeasy Mini Kit, Qiagen, Hilden, Germany). Using a 5 mm steel bead, the tissue was homogenized (2 min at 30 Hz; TissueLyser II, Qiagen), the sample centrifuged and RNA was isolated from the supernatant. RNA isolation was performed for swab samples using the QIAamp Viral RNA Mini Kit and for tissue samples using the RNeasy Mini Kit according to the manufacturer’s instructions (Qiagen). All RNA isolates were then analyzed in duplicates by E-Sarbeco RT-qPCR [37]. Cell culture supernatant isolates of strain hCoV-19/Germany/BY-ChVir-929/2020|EPI_ISL_406862| 2020-01-28 served as positive controls. Samples with a cycle threshold (Ct) value higher than 38 or undetectable were scored negative.

#### 2.2.2. WNV

RNA isolates for WNV RT-qPCR were prepared as described for SARS-CoV-2 and RT-qPCR was performed for the simultaneous detection of both lineages according to Eiden et al. [54]. The target sequence for this RT-qPCR is located in the 5’ untranslated region (5′-UTR) of the WNV genome. RNA isolates of the WNV strains New York 1999 (lineage 1) and Uganda (lineage 2) were included as controls.

### 2.3. Surrogate Virus Neutralization Test (sVNT) for SARS-CoV-2 Antibodies

To analyze potential neutralizing antibodies against SARS-CoV-2, clotted blood samples were centrifuged and serum was pipetted off for serological analysis. Samples were assayed using the SARS-CoV-2 Surrogate Virus Neutralization Test (sVNT) Kit (Genscript, Najing, China) according to manufactures instructions. Briefly, samples were preincubated with horseradish-peroxidase labelled SARS-CoV-2 receptor binding domain (RBD) and subsequently added to a human angiotensin converting enzyme 2 (ACE2)-coated capture plate. After washing, the plate was developed by adding TMB substrate to the plate and after 15 min the reaction was stopped by adding sulfuric acid. Absorption at 450 nm was measured, and after validation check of the plate, inhibition of RBD-binding to ACE2 was calculated in reference to a negative control. Samples with a calculated inhibition greater than 20% were considered positive.

### 2.4. Serological Investigation for Flaviviruses

In total, 205 blood samples underwent a primary serological screening using a commercial blocking ELISA (INgezim West Nile Compac, Ingenasa, Madrid, Spain), where an inhibition percentage (IP) ≥40% was considered positive, >30% to <40% doubtful and ≤30% negative. To confirm and differentiate positive and doubtful ELISA results, reactive samples were also tested in specific virus neutralization tests (VNT) for WNV, USUV and TBEV, as adopted by Seidowski et al. [55] with minor modifications. For the tests, the following virus strains were used: WNV lineage 2 strain Germany (GenBank accession no. MH924836), USUV strain Europe 3 (GenBank accession no. HE599647) and TBEV strain “Neudoerfl” (GenBank accession no. U27495, kindly provided by G. Dobler, Bundeswehr Institute of Microbiology, Munich, Germany). The neutralizing antibody titer (ND_50_) was calculated with the Behrens–Kaerber method [56] and determined as the reciprocal of the serum dilution that inhibited a cytopathogenic effect in >50% of the replicates. The cut-off for the VNTs was set at a 10 (ND_50_ ≥ 10 are positive; ND_50_ < 10 are negative) and differentiation between the flaviviruses was only possible with the presence of a reactive ELISA result and either only a positive result in one of the three VNTs or a four-fold difference between the cross-reacting antibodies [57]. In a few cases, the species-specific antibodies could not be determined by VNT and were, therefore, excluded from the seroprevalence calculations (an infection by an undetermined flavivirus or by multiple flaviviruses). 

## 3. Results

### 3.1. Investigation of Occurence of SARS-CoV-2 in Raccoons and Raccoon Dogs in Germany

**Figure 1:**
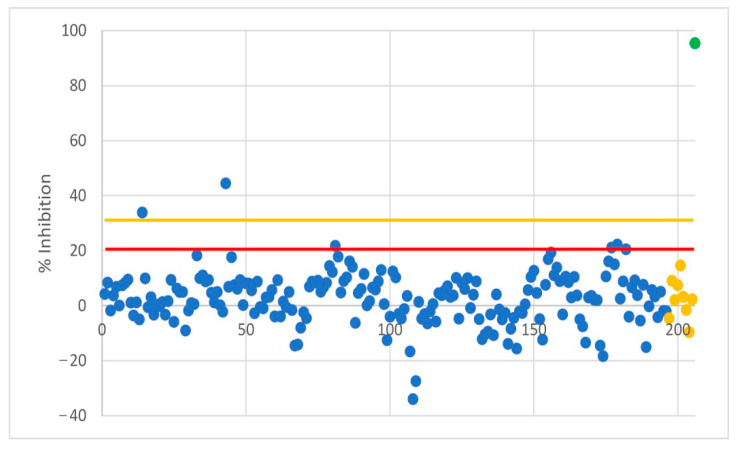
Results of surrogate virus neutralization test (sVNT). Two samples have been found to be positive during the first screening round. They were retested and found to be negative. Red line: Cut−off for reactive samples at 20% inhibition, orange line: Cut−off for positive samples at 30% inhibition. Blue: raccoons; yellow: raccoon dogs; green: mean of positive controls.

For the molecular-biological analysis, samples of 229 raccoons and 11 raccoon dogs (Table 1) were analyzed for SARS-CoV-2 genomic nucleic acids as described in the Material and Methods (Figure 1). For each individuum three samples (Forensic swab L, Virocult^®^ swab and lungs tissue) were assayed. All analyzed swab samples (upper respiratory tract) as well as tissues of the lower respiratory tract were found to be negative for SARS-CoV-2-specific nucleic acids. Thus, no acute or chronic infection with SARS-CoV-2 was detected in the examined animals. In order to verify whether the animals had contact with SARS-CoV-2 in the past, the blood samples of the animals were further examined for the presence of specific neutralizing antibodies.

In the SARS-CoV-2 sVNT (GenScript), a horseradish peroxidase (HRP)-labeled RBD of the spike protein of SARS-CoV-2 binds to ACE2, which in turn is coupled to an ELISA plate. As a result of this binding, an RBD-HRP-ACE2 complex is obtained, which can be detected via the addition of an HRP substrate (TMB). However, if an antibody binds to the RBD in a pre-incubation step, binding to the ACE2 is inhibited and no conversion of the TMB to its soluble end product occurs. In vivo, this process would interfere with the binding of the virus to its host cell. Accordingly, only such inhibitory antibodies are detected in this assay and no antibodies against other domains of the S-protein or other viral proteins.

Serum samples of 196 raccoons and 9 raccoon dogs were used in the assay (Figure 1). The test is considered positive if the inhibition is greater than 30% relative to a negative control and doubtful if the value is between 20% and 30%. Two samples had a value greater than 30% inhibition and both samples were retested and found to be negative. All other samples had an inhibition value less than 20%. The mean of the positive controls from all of the individual tests was 95.3% ± 1.2. No inhibitory antibody could be detected in the sVNT.

Concerning the molecular and serological investigations of SARS-CoV-2, no infection of the German raccoon population with the pandemic coronavirus could be deduced so far.

### 3.2. Incidence of WNV in the Domestic Raccoon Population

WNV-specific RT-qPCR was performed using RNA from blood, as relevant organs (e.g., brain or kidney) were not available. WNV-specific nucleic acids could not be detected in the examined samples. Therefore, concerning flaviviruses, the focus was placed on the serological evaluation of the collected raccoon/raccoon dog blood samples. First, the sera were tested with the commercial flavivirus-specific blocking ELISA. Positive samples were than tested in VNTs for confirmation and differentiation between antibodies against WNV, USUV and TBEV.

**Figure 2:**
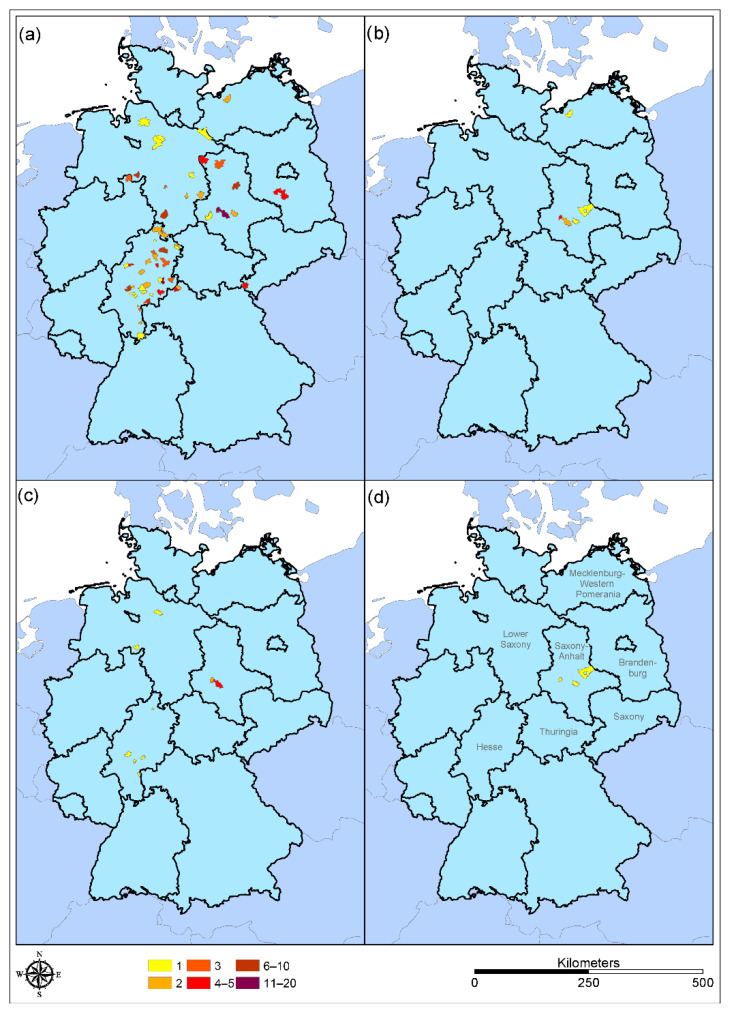
Origin and number of tested samples. (**a**) Sampling raccoon (*Procyon lotor*), (**b**) sampling raccoon dog (*Nyctereutes procyonoides*), (**c**) flavivirus-positive tested raccoon samples (*Procyon lotor*), (**d**) flavivirus-positive tested raccoon dog samples (*Nyctereutes procyonoides*). Number of samples per sampling site are represented by colors, as indicated.

Of the 205 tested blood serum samples (195 raccoons, 10 raccoon dogs), 19 (9.27%) were reactive (positive or doubtful) in the flavivirus-blocking ELISA. Of these, 16 were from raccoons and 3 from raccoon dogs. Subsequent SNTs performed with the reactive samples allowed an estimate of the seroprevalence of WNV (3.6%), USUV (1.5%) and TBEV (0%) for raccoons and a proportional calculation of positive raccoon dogs for WNV (0%), USUV (11.1%) and TBEV (11.1%) in the sample pool examined (Figure 2 and Table 2, Table 3, Table 4 and Table 5). Due to the small sample size for raccoon dogs, it is not possible to make a serious statement about the seroprevalence of the population.

## 4. Discussion

In view of increasing urbanization, the resulting reduction in the habitat of wild animals in so-called industrialized countries, and globalization in conjunction with climate change, it is essential to monitor wild animal populations with regard to zoonotic pathogens to maintain healthy ecological systems [14,15]. Special attention should be paid to zoonotic pathogens that are able to build up reservoirs in which they can further evolve to potentially infect new host species with new traits. In such studies, invasive species have a special position: through immigration, they can bring new pathogens with them, or existing pathogens can encounter new hosts with, for example, new protein structures that may be responsible for altered pathogenesis [23,24]. Two of these neozoa, raccoons and raccoon dogs, were investigated in the present study with respect to two zoonotic pathogens of importance in Germany.

SARS-CoV-2 is the most recent important pathogen threatening not only Germany but the whole world [31,58,59,60,61]. From previous studies with severe acute respiratory syndrome coronavirus 1 (SARS-CoV), it is known that raccoon dogs are susceptible to this coronavirus [62] and pathogenesis experiments showed that they are also susceptible to SARS-CoV-2 [34]. Therefore, it stands to reason that the native wild population of these animals may also carry the virus and form an uncovered reservoir. A second invasive species that is unrelated to the raccoon dog, but has a degree of relationship to the Musteloidea species, which are known to be susceptible to SARS-CoV-2 (mink) and can also return infections to humans, is the raccoon [36]. This species is a good potential host, capable of spreading an anthropozoonotic pathogen within its species, due to its ever-expanding habitat, nocturnal lifestyle and social behavior.

In addition to the currently ubiquitous SARS-CoV-2, WNV infections have been a special challenge in Germany for the past five years. Besides TBEV and USUV, WNV is the third flavivirus that is now endemic in Germany [46,63,64]. Even though there is currently no evidence that raccoons already are a conventional host for WNV, this mosquito-borne arbovirus has the potential to infect a broad range of species, thereby possibly creating a new reservoir species. Occasional infections of other, potential “new reservoir” species may result in heavy virus shedding. Furthermore, since individuals often use the same places for defecation, inter- and intraspecies fecal-oral WNV transmissions seem possible [65].

In the samples analyzed, no SARS-CoV-2 specific nucleic acid could be detected by E-gene based RT-qPCR according to Corman et al. [37]. Although the animals originated from different locations in Northern and Eastern Germany, the main distribution area of the neozoa raccoon and raccoon dog, and the samples were collected over a longer period of time, there appears to be no acute chain of infection within the raccoon population. This is in agreement with a previously published article in which approximately 800 raccoon samples from lungs were also analyzed for the presence of SARS-CoV-2 [66].

The fact that there is no acute infection within the raccoon population is also supported by a lack of neutralizing antibodies in the blood of the animals. Values close to the threshold in the sVNT can be attributed to variations in the quality of the samples. Since the sVNT detects antibodies that are able to inhibit the binding of the RBD to the host cell receptor, this assay is the most suitable for the detection of a former virus infection, also for variants differing from the original strain in the S-protein.

Human health would face a new threat if flaviviruses were to build up a new virus reservoir in the wildlife raccoon population. To date, neither active virus nor genomic RNA from flaviviruses could be isolated from raccoons or raccoon dogs. However, other seroprevalence studies [67,68,69] show that North American as well as Japanese raccoons are positive for antibodies against flaviviruses of the Japanese encephalitis serogroup. This indicates that raccoons occasionally come into contact with the virus, most probably due to the transmission by mosquito vectors which take their blood meal from raccoons as well as birds. Raccoons (and possibly also raccoon dogs) might serve as dead end hosts like other mammals, including humans. In dead end hosts, virus replication is not high enough for a transmission of the virus by vectors or shedding to other hosts. To what extent, however, cross-reactivity between antibodies against the different flaviviruses plays a role for protection against infection with related viruses cannot be clarified yet. Our results from the neutralization tests have shown that reactivity against the other viruses is definitely present and that in certain cases no differentiation between the antibodies against the different flaviviruses is possible.

When comparing the sites at which the raccoon samples were collected with the reports of the notifiable WNV infections, it becomes clear that there is a good correlation: with exception of one animal (Raccoon-B-180), all WNV-positive raccoons were from Saxony-Anhalt, a German federal country with high numbers of WNV infections [70]. Surprisingly, one raccoon hunted in the southwest of Hamburg showed very high reactivity for antibodies against WNV in the SNT. Recently, one horse and one carrion crow (*Corvus corone*) from Hamburg (linear distance < 50 km) have been reported to be positive for WNV [71]. Taken together, these three recent positive findings are indications of a westward spread of WNV in Germany. This is in good accordance with data published by Ziegler et al. for the spread of WNV in 2019 and 2020 in Germany [64] and recent data found at the website of the FLI [72] and in TSIS (Animal Disease Information System [71]). Therefore, it is of extremely valuable importance to conduct health surveillance regarding so-called exotic pathogens, even in species that are not subject to mandatory reporting for WNV, such as horses and birds. One further raccoon from Lower Saxony positive for USUV was hunted in a region in which USUV was detected in the past [64].

Unfortunately, it is not possible to make concrete statements about the seroprevalence in raccoon dogs in this study due to the low number of included samples. However, it should not be overlooked that, similar to the raccoons, seropositive raccoon dogs are also hunted in regions in which WNV-positive cases are regularly reported [39].

The possible contact of the mentioned species with WNV can only be shown indirectly by serological methods. A presence of specific antibodies in the blood of the examined animals suggests a previous infection without being able to make a conclusion about the severity of a disease. Studies have also been performed using RT-qPCR to detect viral genomes, but for these viruses, samples designed for the testing of SARS-CoV-2 were not optimal. Blood and lung samples are not as suitable for the detection of WNV. Samples such as brain or kidney are better suited for this purpose [73], and this will need to be considered in future studies focusing specifically on flaviviruses.

## 5. Conclusions

From the present study, no concrete evidence of SARS-CoV-2 circulation in the German population of raccoons and raccoon dogs can be deduced, not even their susceptibility to the virus. All samples examined are negative both molecularly and serologically. This provides an indication that a reservoir of SARS-CoV-2 is not expected to develop in the raccoon population in the short term. Similarly, the detection of inputs of WNV, USUV and TBEV into the raccoon population is based only on the detection of specific antibodies against these flaviviruses. Detection of WNV genome from the samples was not successful. Thus, reservoir formation in raccoons cannot be demonstrated at this time. 

## Figures and Tables

**Table 1:** Origin of the tested samples. Samples were collected in eight German federal states with focus on Hesse and Saxony-Anhalt. All samples were tested for SARS-CoV-2 by RT-qPCR and sVNT.

German Federal State	Raccoon (*n* = 229)		Raccoon Dog (*n* = 11)	
	*n*	%	*n*	%
Baden-Wurttemberg	4	1.75		
Bavaria	10	4.37	-	-
Brandenburg	4	1.75	-	-
Hesse	104	45.41	-	-
Mecklenburg-Western Pomerania	14	6.11	2	18.18
Lower Saxony	30	13.10	-	-
North-Rhine Westphalia	3	1.31	-	-
Saxony	6	2.62	-	-
Saxony-Anhalt	54	23.58	9	81.82

**Table 2:** Origin of animals found positive in neutralization assays. One animal tested positive for WNV originated from Lower Saxony (bold), all others from Saxony-Anhalt. Another raccoon from Lower Saxony is positive for antibodies against USUV, while WNV antibodies were not detected in this animal. WNV positive raccoon from Lower Saxony is shown in bold. n.d.: No differentiation by neutralization assay possible.

Sample Number	Postal Code	Location	Date	Sex	Age	Land Cover	Positive for
Raccoon-B-023	63500	Seligenstadt (Hesse)	23 February 2021	male	adult	forest	n.d.
Raccoon-B-033	31600	Uchta (Lower Saxony)	5 March 2021	female	adult	farmland	USUV
Raccoon-B-034	34131	Kassel (Hesse)	28 February 2021	male	adult	urban	n.d.
Raccoon-B-120	35510	Butzbach (Hesse)	17 September 2021	female	adult	urban	n.d.
Raccoon-B-125	06458	Hausneindorf (Saxony-Anhalt)	3 September 2021	female	juvenil	forest	n.d.
Raccoon-B-142	06466	Gatersleben (Saxony-Anhalt)	8 October 2021	female	adult	urban	WNV
Raccoon-B-153	61194	Assenheim (Hesse)	7 October 2021	male	adult	urban	USUV
Raccoon-B-155	06466	Gatersleben (Saxony-Anhalt)	14 October 2021	male	juvenil	urban	WNV
Raccoon-B-171	06449	Neu-Königsaue (Saxony-Anhalt)	18 November 2021	female	juvenil	forest	WNV
Raccoon-B-174	06449	Neu-Königsaue (Saxony-Anhalt)	6 November 2021	male	adult	forest	WNV
Raccoon-B-178	06449	Neu-Königsaue (Saxony-Anhalt)	9 January 2022	male	adult	forest	USUV
Raccoon-B-179	06458	Hausneindorf (Saxony-Anhalt)	9 January 2022	female	adult	forest	n.d.
**Raccoon-B-180**	**27389**	**Stemmen (Lower Saxony)**	**14 January 2022**	**male**	**adult**	**farmland**	**WNV**
Raccoon-B-185	63683	Schwickartshausen (Hesse)	30 January 2022	male	adult	forest	n.d.
Raccoon-B-190	06449	Neu-Königsaue (Saxony-Anhalt)	16 February 2022	male	adult	forest	WNV
Raccoon-B-201	06449	Neu-Königsaue (Saxony-Anhalt)	19 March 2022	female	adult	forest	WNV
Raccoon Dog-B-002	39264	Gommern (Saxony-Anhalt)	20 February 2021	male	adult	forest	n.d
Raccoon Dog-B-004	06406	Bernburg (Saxony-Anhalt)	27 February 2021	female	adult	forest	TBEV
Raccoon Dog-B-006	06458	Hausneindorf (Saxony-Anhalt)	19 March 2022	male	juvenil	farmland	USUV

**Table 3:** Estimation of seroprevalence of WNV, USUV and TBEV by differentiation of antibody reactivity of ELISA-reactive samples in neutralization assays.

	WNV	USUV	TBEV
Raccoons	7/195 (3.6%)	3/195 (1.5%)	0/195
Raccoon dogs	0/9	1/9 (11.1%)	1/9 (11.1%)

**Table 4:** Reactive flavivirus-ELISA results of raccoon/raccoon dog serum samples and differentiation between WNV, USUV and TBEV by neutralization tests. Samples were considered positive (POS) by blocking ELISA when the inhibition percentage (IP) was ≥40% and doubtful (DOUBT) by IP > 30% to <40% and IP ≤ 30% as negative (not shown). Positive ELISA and neutralization assay results are shown in bold; questionable ELISA results and cross-reacting antibody titers are also displayed.

	Blocking ELISA		Differentiation by Virus Neutralization Tests		
FLI ID-Number	Inhibition Percentage (IP%)	Result	WNV Antibody Titer (ND_50_)	USUV Antibody Titer (ND_50_)	TBEV Antibody Titer (ND_50_)
1-W-B-142	**93.02**	POS	**1920**	160	<10
1-W-B-155	**94.64**	POS	**2560**	60	<10
1-W-B-171	**91.56**	POS	**120**	<10	15
1-W-B-174	**94.33**	POS	**640**	30	<10
1-W-B-180	**94.22**	POS	**2560**	480	<10
1-W-B-190	**93.15**	POS	**40**	10	<10
1-W-B-201	**94.71**	POS	**960**	20	<10
1-M-B-006	**86.44**	POS	<10	**80**	<10
1-W-B-033	**85.71**	POS	<10	**40**	<10
1-W-B-153	34.87	DOUBT	<10	**80**	<10
1-W-B-178	**44.12**	POS	10	**80**	<10
1-M-B-004	**89.74**	POS	30	15	**120**

**Table 5:** Reactive flavivirus ELISA results from raccoon blood samples where no differentiation was possible between WNV, USUV and TBEV by neutralization assays. Samples were considered positive (POS) by blocking ELISA when the inhibition percentage (IP) was ≥40%, doubtful (DOUBT) when IP > 30% to <40% and negative when IP ≤ 30% (not shown). Positive ELISA and neutralization assay results are shown in bold. As no differentiation was possible via neutralization assays, these serum samples from the raccoons were excluded from the calculation of seroprevalences and were classified as an infection by an undetermined flavivirus or by multiple flaviviruses.

	Blocking ELISA		Differentiation by Virus Neutralization Tests		
FLI ID-Number	Inhibition Percentage (IP%)	Result	WNV Antibody Titer (ND_50_)	USUV Antibody Titer (ND_50_)	TBEV Antibody Titer (ND_50_)
1-W-B-125	**47.27**	POS	480	80	160
1-M-B-002	**43.17**	POS	<10	30	10
1-W-B-023	32.61	DOUBT	<10	<10	<10
1-W-B-034	32.04	DOUBT	<10	20	10
1-W-B-120	33.55	DOUBT	30	60	<10
1-W-B-179	**88.90**	POS	80	30	<10
1-W-B-185	40.24	POS	40	30	<10

## Data Availability

The data presented in this study are available on request from the corresponding author.

## References

[1] Lawson B., Neimanis A., Lavazza A., López-Olvera J.R., Tavernier P., Billinis C., Duff J.P., Mladenov D.T., Rijks J.M., Savić S. (2021). How to Start Up a National Wildlife Health Surveillance Programme. Animals.

[2] Schilling A.-K., Mazzamuto M.V., Romeo C. (2022). A Review of Non-Invasive Sampling in Wildlife Disease and Health Research: What’s New?. Animals.

[3] Cardoso B., García-Bocanegra I., Acevedo P., Cáceres G., Alves P.C., Gortázar C. (2022). Stepping up from wildlife disease surveillance to integrated wildlife monitoring in Europe. Res. Vet. Sci..

[4] Kessler S., Harder T.C., Schwemmle M., Ciminski K. (2021). Influenza A Viruses and Zoonotic Events-Are We Creating Our Own Reservoirs?. Viruses.

[5] Aznar I., Baldinelli F., Papanikolaou A., Stoicescu A., Van der Stede Y. (2021). Annual Report on surveillance for avian influenza in poultry and wild birds in Member States of the European Union in 2020. EFSA J..

[6] Verhagen J.H., Fouchier R.A.M., Lewis N. (2021). Highly Pathogenic Avian Influenza Viruses at the Wild-Domestic Bird Interface in Europe: Future Directions for Research and Surveillance. Viruses.

[7] Frant M.P., Gal-Cisoń A., Bocian Ł., Ziętek-Barszcz A., Niemczuk K., Szczotka-Bochniarz A. (2022). African Swine Fever (ASF) Trend Analysis in Wild Boar in Poland (2014–2020). Animals.

[8] Sauter-Louis C., Conraths F.J., Probst C., Blohm U., Schulz K., Sehl J., Fischer M., Forth J.H., Zani L., Depner K. (2021). African Swine Fever in Wild Boar in Europe-A Review. Viruses.

[9] Blome S., Franzke K., Beer M. (2020). African swine fever-A review of current knowledge. Virus Res..

[10] Garnier R., Graham A.L. (2014). Insights from Parasite-Specific Serological Tools in Eco-Immunology. Am. Zool..

[11] Nijman V. (2021). Illegal and Legal Wildlife Trade Spreads Zoonotic Diseases. Trends Parasitol..

[12] Bezerra-Santos M.A., Mendoza-Roldan J.A., Thompson R.C.A., Dantas-Torres F., Otranto D. (2021). Legal versus Illegal Wildlife Trade: Zoonotic Disease Risks. Trends Parasitol..

[13] Lafferty K.D. (2009). The ecology of climate change and infectious diseases. Ecology.

[14] Schell C.J., Stanton L.A., Young J.K., Angeloni L.M., Lambert J.E., Breck S.W., Murray M.H. (2021). The evolutionary consequences of human-wildlife conflict in cities. Evol. Appl..

[15] König H.J., Kiffner C., Kramer-Schadt S., Fürst C., Keuling O., Ford A.T. (2020). Human-wildlife coexistence in a changing world. Conserv. Biol..

[16] Castillo-Contreras R., Mentaberre G., Fernandez Aguilar X., Conejero C., Colom-Cadena A., Ráez-Bravo A., González-Crespo C., Espunyes J., Lavín S., López-Olvera J.R. (2021). Wild boar in the city: Phenotypic responses to urbanisation. Sci. Total Environ..

[17] Munnink B.B.O., Sikkema R.S., Nieuwenhuijse D.F., Molenaar R.J., Munger E., Molenkamp R., Van Der Spek A., Tolsma P., Rietveld A., Brouwer M. (2021). Transmission of SARS-CoV-2 on mink farms between humans and mink and back to humans. Science.

[18] Banerjee A., Mossman K., Baker M.L. (2021). Zooanthroponotic potential of SARS-CoV-2 and implications of reintroduction into human populations. Cell Host Microbe.

[19] Kock R., Michel A.L., Yeboah-Manu D., Azhar E.I., Torrelles J.B., Cadmus S.I., Brunton L., Chakaya J.M., Marais B., Mboera L. (2021). Zoonotic Tuberculosis-The Changing Landscape. Int. J. Infect. Dis..

[20] Shivaprakash K.N., Sen S., Paul S., Kiesecker J.M., Bawa K.S. (2021). Mammals, wildlife trade, and the next global pandemic. Curr. Biol..

[21] Fagre A.C., Cohen L.E., Eskew E.A., Farrell M., Glennon E., Joseph M.B., Frank H.K., Ryan S.J., Carlson C.J., Albery G.F. (2022). Assessing the risk of human-to-wildlife pathogen transmission for conservation and public health. Ecol. Lett..

[22] Račnik J., Kočevar A., Slavec B., Korva M., Rus K.R., Zakotnik S., Zorec T.M., Poljak M., Matko M., Rojs O.Z. (2021). Transmission of SARS-CoV-2 from Human to Domestic Ferret. Emerg. Infect. Dis..

[23] Rentería-Solís Z.M., Hamedy A., Michler F.-U., Michler B.A., Lücker E., Stier N., Wibbelt G., Riehn K. (2013). *Alaria alata* mesocercariae in raccoons (*Procyon lotor*) in Germany. Parasitol. Res..

[24] Fischer M.L., Sullivan M.J.P., Greiser G., Guerrero-Casado J., Heddergott M., Hohmann U., Keuling O., Lang J., Martin I., Michler F.-U. (2016). Assessing and predicting the spread of non-native raccoons in Germany using hunting bag data and dispersal weighted models. Biol. Invasions.

[25] Bharti A.R., Nally J.E., Ricaldi J.N., Matthias M.A., Diaz M.M., Lovett M.A., Levett P.N., Gilman R.H., Willig M.R., Gotuzzo E. (2003). Leptospirosis: A zoonotic disease of global importance. Lancet Infect. Dis..

[26] Beltrán-Beck B., García F.J., Gortázar C. (2012). Raccoons in Europe: Disease hazards due to the establishment of an invasive species. Eur. J. Wildl. Res..

[27] Michelitsch A., Wernike K., Ulrich L., Mettenleiter T.C., Beer M. (2021). SARS-CoV-2 in animals: From potential hosts to animal models. Adv Virus Res.

[28] Holmes E.C., Goldstein S.A., Rasmussen A.L., Robertson D.L., Crits-Christoph A., Wertheim J.O., Anthony S.J., Barclay W.S., Boni M.F., Doherty P.C. (2021). The origins of SARS-CoV-2: A critical review. Cell.

[29] Rutherford C., Kafle P., Soos C., Epp T., Bradford L., Jenkins E. (2022). Investigating SARS-CoV-2 Susceptibility in Animal Species: A Scoping Review. Environ. Health Insights.

[30] Shi J., Wen Z., Zhong G., Yang H., Wang C., Huang B., Liu R., He X., Shuai L., Sun Z. (2020). Susceptibility of ferrets, cats, dogs, and other domesticated animals to SARS-coronavirus 2. Science.

[31] Guo Y.-R., Cao Q.-D., Hong Z.-S., Tan Y.-Y., Chen S.-D., Jin H.-J., Tan K.-S., Wang D.-Y., Yan Y. (2020). The origin, transmission and clinical therapies on coronavirus disease 2019 (COVID-19) outbreak–an update on the status. Mil. Med. Res..

[32] World Health Organization (2022). Weekly Epidemiological Update on COVID-19.

[33] Aguirre A.A., Catherina R., Frye H., Shelley L. (2020). Illicit wildlife trade, wet markets, and COVID-19: Preventing future pandemics. World Med. Health Policy.

[34] Freuling C.M., Breithaupt A., Müller T., Sehl J., Balkema-Buschmann A., Rissmann M., Klein A., Wylezich C., Hoper D., Wernike K. (2020). Susceptibility of raccoon dogs for experimental SARS-CoV-2 infection. Emerg. Infect. Dis..

[35] Chueca L.J., Kochmann J., Schell T., Greve C., Janke A., Pfenninger M., Klimpel S. (2021). De novo Genome Assembly of the Raccoon Dog (*Nyctereutes procyonoides*). Front. Genet..

[36] Francisco R., Hernandez S.M., Mead D.G., Adcock K.G., Burke S.C., Nemeth N.M., Yabsley M.J. (2021). Experimental susceptibility of North American raccoons (*Procyon lotor*) and striped skunks (*Mephitis mephitis*) to SARS-CoV-2. Front. Vet. Sci..

[37] Corman V.M., Landt O., Kaiser M., Molenkamp R., Meijer A., Chu D.K.W., Bleicker T., Brunink S., Schneider J., Schmidt M.L. (2020). Detection of 2019 novel coronavirus (2019-nCoV) by real-time RT-PCR. Euro. Surveill..

[38] Santos P.D., Michel F., Wylezich C., Hoper D., Keller M., Holicki C.M., Szentiks C.A., Eiden M., Muluneh A., Neubauer-Juric A. (2021). Co-infections: Simultaneous detections of West Nile virus and Usutu virus in birds from Germany. Transbound. Emerg. Dis..

[39] Ziegler U., Santos P.D., Groschup M.H., Hattendorf C., Eiden M., Hoper D., Eisermann P., Keller M., Michel F., Klopfleisch R. (2020). West Nile Virus Epidemic in Germany Triggered by Epizootic Emergence, 2019. Viruses.

[40] Bakonyi T., Haussig J.M. (2020). West Nile virus keeps on moving up in Europe. Eurosurveillance.

[41] Klaus C., Horugel U., Hoffmann B., Beer M. (2013). Tick-borne encephalitis virus (TBEV) infection in horses: Clinical and laboratory findings and epidemiological investigations. Vet. Microbiol..

[42] Pulkkinen L.I.A., Butcher S.J., Anastasina M. (2018). Tick-Borne Encephalitis Virus: A Structural View. Viruses.

[43] Lindquist L., Vapalahti O. (2008). Tick-borne encephalitis. Lancet.

[44] Sinnecker H. (1960). Zeckenencephalitis in Deutschland. Zbl. Bact. Orig..

[45] Ziegler U., Lühken R., Markus K., Cadar D., van der Grinten E., Michel F., Albrecht K., Eiden M., Rinder M., Lachmann L. (2018). West Nile virus epizootic in Germany, 2018. Elsevier.

[46] Michel F., Sieg M., Fischer D., Keller M., Eiden M., Reuschel M., Schmidt V., Schwehn R., Rinder M., Urbaniak S. (2019). Evidence for West Nile Virus and Usutu Virus Infections in Wild and Resident Birds in Germany, 2017 and 2018. Viruses.

[47] Jost H., Bialonski A., Maus D., Sambri V., Eiden M., Groschup M.H., Gunther S., Becker N., Schmidt-Chanasit J. (2011). Isolation of usutu virus in Germany. Am. J. Trop. Med. Hyg..

[48] Becker N., Jost H., Ziegler U., Eiden M., Hoper D., Emmerich P., Fichet-Calvet E., Ehichioya D.U., Czajka C., Gabriel M. (2012). Epizootic emergence of Usutu virus in wild and captive birds in Germany. PLoS ONE.

[49] Ziegler U., Jost H., Muller K., Fischer D., Rinder M., Tietze D.T., Danner K.J., Becker N., Skuballa J., Hamann H.P. (2015). Epidemic Spread of Usutu Virus in Southwest Germany in 2011 to 2013 and Monitoring of Wild Birds for Usutu and West Nile Viruses. Vector-Borne Zoonotic Dis..

[50] Cadar D., Bosch S., Jost H., Borstler J., Garigliany M.M., Becker N., Schmidt-Chanasit J. (2015). Putative Lineage of Novel African Usutu Virus, Central Europe. Emerg. Infect. Dis..

[51] Ashraf U., Ye J., Ruan X., Wan S., Zhu B., Cao S. (2015). Usutu virus: An emerging flavivirus in Europe. Viruses.

[52] Heinz F.X., Stiasny K. (2012). Flaviviruses and their antigenic structure. J. Clin. Virol..

[53] Beck C., Lowenski S., Durand B., Bahuon C., Zientara S., Lecollinet S. (2017). Improved reliability of serological tools for the diagnosis of West Nile fever in horses within Europe. PLoS Negl. Trop. Dis..

[54] Eiden M., Vina-Rodriguez A., Hoffmann B., Ziegler U., Groschup M.H. (2010). Two new real-time quantitative reverse transcription polymerase chain reaction assays with unique target sites for the specific and sensitive detection of lineages 1 and 2 West Nile virus strains. J. Vet. Diagn. Investig..

[55] Seidowski D., Ziegler U., von Ronn J.A., Muller K., Huppop K., Muller T., Freuling C., Muhle R.U., Nowotny N., Ulrich R.G. (2010). West Nile virus monitoring of migratory and resident birds in Germany. Vector-Borne Zoonotic Dis..

[56] Mayr A., Bachmann P.A., Bibrack B., Wittmann G., Mayr A., Bachmann P.A., Bibrack B., Wittmann G. (1977). Neutralisationstest. Virologische Arbeitsmethoden, Band II (Serologie).

[57] Yeh J.Y., Lee J.H., Park J.Y., Seo H.J., Moon J.S., Cho I.S., Kim H.P., Yang Y.J., Ahn K.M., Kyung S.G. (2012). A diagnostic algorithm to serologically differentiate West Nile virus from Japanese encephalitis virus infections and its validation in field surveillance of poultry and horses. Vector-Borne Zoonotic Dis..

[58] Zhu N., Zhang D., Wang W., Li X., Yang B., Song J., Zhao X., Huang B., Shi W., Lu R. (2020). A Novel Coronavirus from Patients with Pneumonia in China, 2019. N. Engl. J. Med..

[59] Chan J.F.-W., Yuan S., Kok K.-H., To K.K.-W., Chu H., Yang J., Xing F., Liu J., Yip C.C.-Y., Poon R.W.-S. (2020). A familial cluster of pneumonia associated with the 2019 novel coronavirus indicating person-to-person transmission: A study of a family cluster. Lancet.

[60] Boehm E., Kronig I., Neher R.A., Eckerle I., Vetter P., Kaiser L., Geneva Center for Emerging Viral Diseases (2021). Novel SARS-CoV-2 variants: The pandemics within the pandemic. Clin. Microbiol. Infect..

[61] World Health Organization, Coronavirus Disease (COVID-19) Weekly Epidemiological Update and Weekly Operational Update. https://www.who.int/emergencies/diseases/novel-coronavirus-2019/situation-reports.

[62] Guan Y., Zheng B.J., He Y.Q., Liu X.L., Zhuang Z.X., Cheung C.L., Luo S.W., Li P.H., Zhang L.J., Guan Y.J. (2003). Isolation and Characterization of Viruses Related to the SARS Coronavirus from Animals in Southern China. Science.

[63] Ziegler U., Angenvoort J., Klaus C., Nagel-Kohl U., Sauerwald C., Thalheim S., Horner S., Braun B., Kenklies S., Tyczka J. (2013). Use of competition ELISA for monitoring of West Nile virus infections in horses in Germany. Int. J. Environ. Res. Public Health.

[64] Ziegler U., Bergmann F., Fischer D., Müller K., Holicki C.M., Sadeghi B., Sieg M., Keller M., Schwehn R., Reuschel M. (2022). Spread of West Nile Virus and Usutu Virus in the German Bird Population, 2019–2020. Microorganisms.

[65] Root J.J., Bentler K.T., Nemeth N.M., Gidlewski T., Spraker T.R., Franklin A.B. (2010). Experimental infection of raccoons (*Procyon lotor*) with West Nile virus. Am. J. Trop. Med. Hyg..

[66] Hagag I.T., Langner T., Groschup M.H., Keller M. (2022). Molecular surveillance revealed no SARS-CoV-2 spillovers to raccoons (*Procyon lotor*) in four German federal states. Eur. J. Wildl. Res..

[67] Ohno Y., Sato H., Suzuki K., Yokoyama M., Uni S., Shibasaki T., Sashika M., Inokuma H., Kai K., Maeda K. (2009). Detection of antibodies against Japanese encephalitis virus in raccoons, raccoon dogs and wild boars in Japan. J. Vet. Med. Sci..

[68] Blitvich B.J., Juarez L.I., Tucker B.J., Rowley W.A., Platt K.B. (2009). Antibodies to West Nile virus in raccoons and other wild peridomestic mammals in Iowa. J. Wildl. Dis..

[69] Bentler K.T., Hall J.S., Root J.J., Klenk K., Schmit B., Blackwell B.F., Ramey P.C., Clark L. (2007). Serologic evidence of West Nile virus exposure in North American mesopredators. Am. J. Trop. Med. Hyg..

[70] Ganzenberg S., Sieg M., Ziegler U., Pfeffer M., Vahlenkamp T.W., Horugel U., Groschup M.H., Lohmann K.L. (2022). Seroprevalence and Risk Factors for Equine West Nile Virus Infections in Eastern Germany, 2020. Viruses.

[71] Friedrich-Loeffler-Institut, Federal Research Institute for Animal Health TSIS-TierSeuchenInformationsSystem. https://tsis.fli.de/Reports/Info_SO.aspx?ts=416&guid=ff9aa17d-b468-4fad-926a-ce9a1745a1d0.

[72] Friedrich-Loeffler-Institut, Federal Research Institute for Animal Health West-Nil-Virus–Aktueller Stand: Bisher 38 Bestätigte Infektionen bei Pferden und Vögeln in Deutschland. https://www.fli.de/de/aktuelles/kurznachrichten/neues-einzelansicht/aktueller-stand-bisher-38-bestaetigte-west-nil-virus-infektionen-bei-pferden-und-voegeln-in-deutschland/.

[73] Eloit M., OIE, World Organisation for Animal Health (2022). Chapter 8.20.-West Nile Fever. Terrestrial Animal Health Code.

